# Management and prognosis of patients with cancer of unknown primary: 20 years of experience

**DOI:** 10.55730/1300-0144.5741

**Published:** 2023-11-11

**Authors:** Murat BARDAKÇI, Efnan ALGIN, Büşra DÜĞEROĞLU, Öznur Bal, Fahriye Tuğba KÖŞ, Doğan UNCU

**Affiliations:** 1 Division of Oncology, Department of Internal Medicine, University of Health Sciences, Ankara City Hospital, Ankara, Turkiye; 2 Department of Internal Medicine, University of Health Sciences, Ankara City Hospital, Ankara, Turkiye

**Keywords:** Cancer of unknown primary, cholestasis, lung metastasis, chemotherapy, prognosis

## Abstract

**Background/aim:**

Cancer of unknown primary (CUP) is a difficult clinical entity to manage. The aim of the study was to investigate the sociodemographic and pathological characteristics, treatment options, and factors affecting overall survival (OS) in CUP patients whose primary tumor was not detected during follow-up.

**Materials and methods:**

A total of 243 CUP patients whose primary tumors could not be detected during follow-up were included in the study. Their demographic characteristics, survival outcomes, and prognostic factors were investigated.

**Results:**

Of the 243 patients included in this study, 61.7% were male and 38.3% were female, and the median age was 61 (range: 19–90) years. The most common histological type was adenocarcinoma (79%). The median follow-up time of the patients was 30.3 months (95% CI: 11.4–49.3), the median OS time was 9.1 months (95% CI: 7.2–11.0), and 72.4% of the patients received at least 1 line of chemotherapy (CT). The difference in survival between the patients who did and did not receive CT was statistically significant (median OS: 10.1 vs. 4.2 months, p = 0.003). According to the multivariate analysis, the presence of cholestasis (HR: 0.48, 95% CI: 0.29–0.79, p = 0.004), lung metastasis (HR: 0.69, 95% CI: 0.51–0.95, p = 0.001), second-line chemotherapy (HR: 1.69, 95% CI: 1.14–2.49, p < 0.001), and Eastern Cooperative Oncology Group (ECOG) performance status (HR: 0.20, 95% CI: 0.10–0.40, p < 0.001) were independent prognostic factors influencing OS.

**Conclusion:**

CUP patients who receive multiple lines of chemotherapy tend to have longer survival. This is the first study to report cholestasis as a prognostic factor in CUP patients. In addition, the presence of lung metastases, not receiving second-line chemotherapy, and ECOG performance status (≥2) were found to be independent poor prognostic factors.

## 1. Introduction

Approximately one in three patients diagnosed with cancer has distant metastases at the time of diagnosis. In most cases, primary tumors and metastases are defined at the same time. However, despite extensive diagnostic examinations, it is not always possible to identify the primary tumor focus. In this event, patients are diagnosed with cancer of unknown primary (CUP). It has been reported that the primary tumor is detected before death in less than 20% of CUP patients. On the other hand, the primary tumor detection rate is approximately 30% even in autopsy series [1], and it has been reported that this rate has increased to 40% with the widespread use of advanced imaging methods in recent years [2].

CUP is a common disease with an incidence of 3%–5% among other epithelial tumors [1]. At the time of diagnosis, approximately 10% of CUP patients have metastases only in the lymph nodes; the remaining patients have visceral organ involvement [3]. Historically, the median overall survival (OS) time is less than 1 year [4]. The histological type and location of metastases have prognostic importance, with the shortest OS time of 2 months reported in CUP patients with liver metastases [5]. In general, 20% of CUP patients belong to the favorable prognostic group, whereas the remaining 80% are in the unfavorable CUP group, which is characterized by poor survival outcomes [6]. While local treatment options are available in addition to systemic treatments in the favorable group, patients in the unfavorable group are generally treated with empirical chemotherapy [7].

The aim of the present study was to determine the demographic features, treatment approaches, and prognostic factors affecting the survival of CUP patients whose primary tumors could not be detected during follow-up.

## 2. Material and methods

### 2.1. Patients

This retrospective review was conducted on 828 patients who presented to Ankara Numune Training and Research Hospital and Ankara City Hospital with the diagnosis of CUP between March 2002 and May 2021. The inclusion criteria were as follows: 1) age at diagnosis ≥18 years; 2) no previous history of malignancy; 3) primary tumor site was not found on follow-up; and 4) histologically proven malignant epithelial tumors including adenocarcinoma, squamous cell carcinoma (SCC), and undifferentiated carcinoma. Consequently, 243 patients with CUP whose primary tumor could not be detected during follow-up were enrolled in the study. The demographic characteristics of the patients, treatment modalities, and treatment outcomes were recorded. Survival rates and prognostic factors were investigated. All of the patients were grouped according to the median age (≤61/>61), sex (male/female), Eastern Cooperative Oncology Group (ECOG) performance status (0–1/≥2), cholestasis status (yes/no), histological type (adenocarcinoma/undifferentiated carcinoma/SCC), number of metastatic sites (1–2/≥3), lymph node metastasis (yes/no), liver metastasis (yes/no), lung metastasis (yes/no), bone metastasis (yes/no), brain metastasis (yes/no), peritoneal metastasis (yes/no), adrenal gland metastasis (yes/no), first-line chemotherapy (yes/no), second-line chemotherapy (yes/no/unknown), third-line chemotherapy (yes/no/unknown), chemoembolization (yes/no), radiofrequency ablation (yes/no). Cholestasis was defined as follows: in patients with liver metastases with a direct bilirubin level >0.4 mg/dL, other causes of hyperbilirubinemia such as cirrhosis, biliary tract stones, autoimmune, and genetic diseases as well as external compression excluding metastases were excluded. After diagnosis, 27.6% of the patients could not receive chemotherapy due to advanced age, additional comorbidities, and ECOG performance status. At the time of the detection of liver metastasis, cholestasis was defined as present if the serum direct bilirubin level was >0.4 mg/dL after excluding all other possibilities causing hyperbilirubinemia, including drugs, cholelithiasis, primary biliary cirrhosis, primary sclerosing cholangitis, extrahepatic mass compression (gallbladder tumor, pancreatic and or duodenal tumor or cysts), and liver parenchymal disease based on radiologic and laboratory findings.

### 2.2. Diagnostic tests

The patients were investigated in search of a primary site before being classified as CUP. The imaging techniques included abdominal ultrasound, computed tomography (CT) scans of the chest and abdomen, magnetic resonance imaging of the abdomen, positron emission tomography (PET)-CT, mammography, gastroscopy, colonoscopy, and bronchoscopy. Serum tumor markers and immunohistochemical staining on histopathological evaluation were other diagnostic tools used. As a result of the diagnostic tests performed on the patients, the primary tumor site could not be identified in the study population, neither at the time of diagnosis nor during follow-up. Primary molecular analyses (HER-2 for breast cancer, EGFR/ALK/ROS1/PD-L1 for lung cancer, MSI/KRAS/NRAS/BRAF for gastrointestinal cancer) were performed in patients according to clinical characteristics, but next-generation sequencing (NGS) or RNA-based large panel could not be studied routinely. Patients with positive specific molecular profiles were excluded from the study.

### 2.3. Statistical analysis

All statistical analyses was performed using IBM SPSS Statistics for Windows 22.0 (IBM Corp., Armonk, NY, USA. Survival rates were calculated using Kaplan–Meier survival analysis and compared using the log-rank test. OS was defined as the time interval from the date of histopathologic diagnosis by biopsy until death due to any reason or the last follow-up. The log-rank test was used to determine the clinicopathologic variables of the CUP patients related to survival. The Cox proportional hazards model was performed for multivariable analysis. The adjusted hazard ratios (HRs), 95% confidence intervals (CIs), and corresponding p-values were recorded. p < 0.05 was considered statistically significant. Progression-free survival (PFS) was calculated from the initiation of the first treatment up to the date of confirmed disease progression (clinical or radiologically) or death from any cause or last follow-up, whichever occurred first.

## 3. Results

### 3.1. Patient characteristics

The median age of the patients was 61 (ranging: 19–90) years and the majority were male (male/female: 150/93). The most common biopsy site was the liver (57.6%) and adenocarcinoma was the most common histological subtype (79%). The most common metastatic sites were the lymph nodes (81.5%), liver (71.6%), lungs (39.5%), bones (29.2%), peritoneum (21.4%), brain (11.5%), and surrenal gland (9.9%). Cholestasis was present in 9.5% of the patients. The demographic and pathological features of the patients are summarized in Table 1.

Of the 243 patients, 176 (72.4%) received chemotherapy. The most frequently used chemotherapy regimen in first-line treatment was the gemcitabine/cisplatin combination at a rate of 49.4%. Only 50 patients (20.6%) received second-line chemotherapy and the most commonly used regimens were oxaliplatin-containing combinations (42.0%), and 19 patients (7.8%) were managed with third-line chemotherapy. In addition to chemotherapy, 18 patients received transarterial chemoembolization (TACE) and 3 received radiofrequency ablation (RFA). Details of the treatments are summarized in Table 2.

### 3.2. Survival analysis and treatment effect

**Figure 1:**
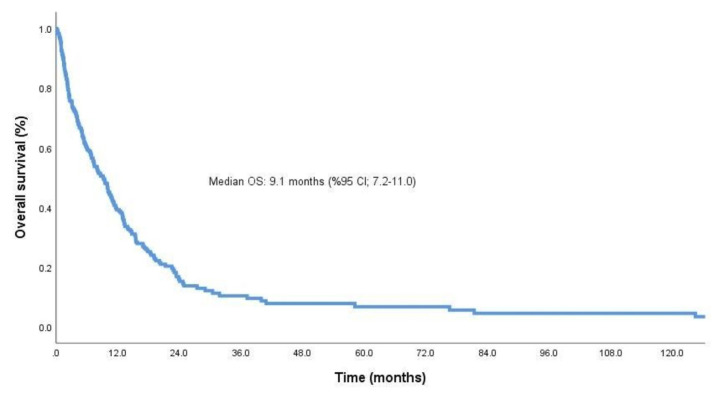
Kaplan–Meier curves for OS in the patients.

The median follow-up time of the patients was 30.3 months (95% CI: 11.4–49.3) and the median OS time was 9.1 months (95% CI: 7.2–11.0) (Figure 1). The median OS time was 12.5 months (95% CI: 9.0–16.0) in patients with metastases at a single site, 7.4 months (95% CI: 4.5–10.2) in patients with metastases at 2 sites, and 6.8 months (95% CI: 3.1–10.4) in patients with metastases at 3 or more sites. Although survival decreased with an increasing number of metastatic sites, the decrease was not statistically significant (p = 0.07).

**Figure 2:**
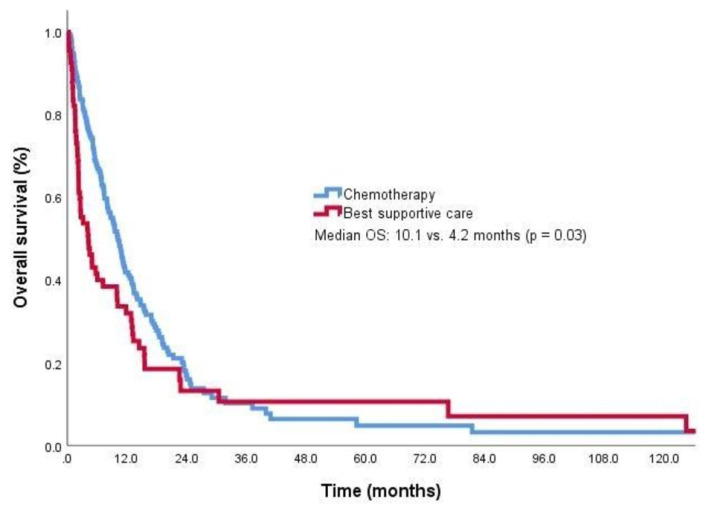
Kaplan–Meier curves for OS according to the chemotherapy status of the CUP patients.

The median OS time was 10.1 months (95% CI: 8.3–11.9) in patients who received at least 1 line of chemotherapy and 4.2 months (95% CI: 1.6–6.7) in those who did not, and the difference was statistically significant (p = 0.03) (Figure 2). The survival results of the patients according to the first-line chemotherapy regimens are shown in Table 3. Longer survival was observed in the patients who received 2 and 3 lines of chemotherapy than in those who received only 1 line of chemotherapy. The median OS time was 16.8 months (95% CI: 12.4–21.3) in patients who received second-line chemotherapy and 7.1 months (95% CI: 5.2–9.1) in those who did not, and the 9.7-month survival difference was statistically significant (p < 0.001). The median OS time was 27.4 months (95% CI: 11.3–43.5) in patients who received third-line chemotherapy and 7.9 months (95% CI: 5.1–10.7) in those who did not, and the difference was statistically significant (p = 0.004). Locoregional treatments (TACE and RFA) did not make a significant difference in terms of survival.

### 3.3. Prognostic factors

Prognostic factors affecting survival in CUP patients were investigated herein. According to the univariate analysis, the ECOG performance status (p < 0.001), cholestasis (p = 0.001), liver metastasis (p = 0.05), lung metastasis (p = 0.002), bone metastasis (p = 0.02), and receiving at least 1 line of chemotherapy (p = 0.03), second-line chemotherapy (p < 0.001), and third-line chemotherapy (p = 0.004) were associated with OS. When these statistically significant variables were evaluated in the Cox regression analysis, the presence of cholestasis (HR: 0.48, 95% CI: 0.29–0.79, p = 0.004), lung metastasis (HR: 0.69, 95% CI: 0.51–0.95, p = 0.001), second-line chemotherapy (HR: 1.69, 95% CI: 1.14–2.49, p < 0.001), and ECOG performance status (HR: 0.20, 95% CI: 0.10–0.40, p < 0.001) were independent prognostic factors affecting OS (Table 4).

## 4. Discussion

CUP is a heterogeneous group of metastatic cancers with a high incidence among all new cancer cases and each is assumed to have different biological characteristics [8]. Management of these cancers requires a comprehensive physical examination, focused imaging, and pathological examination [9]. While the median age at diagnosis is 60 and over, it is reported to be more common in males [10,11]. The most common histologic type is adenocarcinoma, with rates ranging from 60% to 80%. Other epithelial histological types include undifferentiated carcinoma, SCC, and neuroendocrine carcinoma [10,12–14]. In the current study, the median age at diagnosis was 61 years and males (61.7%) were the dominant sex. As in the literature, the most common histological type was adenocarcinoma (78.6%).

CUP are often located in the lymph nodes, liver, lungs, and bones [12,13]. CUP with solitary metastases accounts for only 15%–25% of all cases, while disseminated metastases occur in 75%–85% of cases [10]. In the present study, 72% of the patients had metastatic involvement in more than one area. The most common sites of metastases were the lymph nodes and the liver, in line with the literature.

The median OS time of CUP patients ranges from 6 to 9 months [15]. In addition to the uncertainty of diagnostic tests and treatment modalities, the poor prognosis may be related to the unknown nature of this group of tumors. It is known that prognosis is usually improved if a possible primary tumor is detected, and appropriate treatment is applicable. Moreover, while CUP patients are treated empirically, treatment approaches for certain types of advanced cancer continue to evolve. Therefore, recent studies have focused on molecular gene expression profiling to predict the primary origin and apply specific treatments [16,17]. Hainsworth et al. investigated the effect of molecular tumor profiling on survival in CUP patients prospectively. Although they found a statistically significant survival difference between the patients who received assay-directed site-specific chemotherapy regimens and empiric CUP regimens, the median survival time was also only 12.5 months [18]. In the present study, the median OS time was 9.1 months, consistent with the literature.

In different studies, various prognostic factors were defined, such as age, sex, performance status, weight loss, clinical presentation, localization and extent of tumor, number of metastatic sites, histological type, biochemical parameters (serum albumin, alkaline phosphatase, neutrophil-to-lymphocyte ratio), and serum tumor markers [1,11,14,19,20]. Due to the retrospective design of the current study, it was not possible to evaluate all of these parameters defined in the literature. It was found herein that the ECOG performance status, cholestasis, liver metastasis, lung metastasis, bone metastasis, and receiving at least 1 line of chemotherapy, second-line chemotherapy, and third-line chemotherapy were associated with OS. However, the multivariate analysis revealed that only the presence of cholestasis, lung metastases, not receiving second-line chemotherapy, and the ECOG performance status (≥2) were independent poor prognostic factors for OS. Among the results herein, the relationship between cholestasis and survival is of great importance. The median OS time of the patients with cholestasis in the current study was only 2.5 months. To the best of our knowledge, the present study is the first to report cholestasis as a prognostic factor.

On the other hand, some well-known factors, including the number of metastatic sites, liver metastasis, and histologic types, were found to be unrelated to survival. It is our opinion that the reason for these discordant results is the inadequate distribution of the patient groups as a result of the retrospective study design. The prognostic significance of the number of metastatic sites has been demonstrated in many studies [1,11,19]. In a study of 265 patients with CUP, it was reported that the median OS time decreased to 1.6 months in patients with the involvement of 2 or more metastatic sites [20]. As expected, survival decreased as the number of metastatic sites increased in the present study. However, it did not reach statistical significance. In addition, SCC has been reported as the histological type with the best survival, regardless of age and treatment [21,22]; however, it was not possible to demonstrate any difference in survival between the histological types in the current study. This was probably because the rate of SCC histology was only 3.7%. Finally, the presence of liver metastases is known to be an unfavorable prognostic factor [23,24]. Although the prognostic significance of liver metastases in the univariate analysis was demonstrated herein, it did not remain statistically significant in the multivariate analysis.

In general, chemotherapy prolongs the survival of CUP patients [11,13,14]. In a study conducted with 179 CUP patients, 75.4% received chemotherapy and the OS time of the chemotherapy group was significantly better than that of the group that did not receive it (9.2 vs. 1.6 months, p < 0.001) [25]. The present study results are consistent with the literature in terms of the survival outcomes (10.1 vs. 4.2 months, p = 0.03) and the rate of patients who received chemotherapy (72.4%).

Many chemotherapy regimens have been tried in order to improve survival. Gemcitabine/platinum combinations were the most commonly used regimen, as in the current study. Other options include taxanes, irinotecan, and etoposide. However, none of these are superior in terms of survival [26–29]. The number of chemotherapy lines may be more important for survival than the content of the chemotherapy regimen. In this regard, the present study results revealed that survival improved as the number of chemotherapy lines increased. Given the poor prognosis of the disease, the number of patients who were able to receive multiple lines of chemotherapy was low in the current study. However, receiving second-line chemotherapy was an independent prognostic factor affecting OS.

As mentioned previously, the main limitation of the present study was the retrospective design. Other limitations include the heterogeneity of the patients’ general characteristics and the chemotherapy regimens. It was not possible to include data on targeted molecular therapy or molecular profiling because the study period predated the more widespread use of molecular testing. The lack of access to these tests at the time of patient enrollment limited the ability to investigate the potential impact of molecular markers or genetic alterations on prognosis and treatment outcomes.

## 5. Conclusion

The present study demonstrated that cholestasis, lung metastasis, second-line chemotherapy, and the ECOG performance status are independent prognostic factors for OS in CUP patients. To the best of our knowledge, this is the first report establishing a relationship between cholestasis and survival in CUP patients. In addition, patients who were able to receive multiple lines of chemotherapy tended to have longer survival. Considering the unknown behavior of the disease, these factors are important in guiding the management of patients with CUP.

## Figures and Tables

**Table 1:** Sociodemographic and pathological characteristics of the patients and the treatments applied.

	N: 243 (%)
Age* (years)	61 (19–90)
Sex	
Male	150 (61.7)
Female	93 (38.3)
ECOG†	
0–1	111 (45.7)
≥2	132 (54.3)
Biopsy location	
Liver	140 (57.6)
Bone-bone marrow	18 (7.4)
Lymph node	17 (7.0)
Lung	14 (5.8)
Brain	11 (4.5)
Peritoneum	11 (4.5)
Cytology (ascites, pleural, pericardial)	9 (3.7)
Others	23 (9.5)
Histological type	
Adenocarcinoma	192 (79.0)
Undifferentiated carcinoma	42 (17.3)
SCC	9 (3.7)
Presence of cholestasis	23 (9.5)
Lymph node metastasis	198 (81.5)
Liver metastasis	174 (71.6)
Lung metastasis	96 (39.5)
Bone metastasis	71 (29.2)
Peritoneal metastasis	52 (21.4)
Brain metastasis	28 (11.5)
Adrenal gland metastasis	24 (9.9)
Serum tumor markers*	
CA 19-9 (U/mL)	45.3 (0.5–129128.4)
CEA (ng/mL)	4.3 (0.5–10000.0)
CA 125 (U/mL)	87.5 (3.3–6223.9)
CA 15-3 (U/mL)	29.4 (3.9–942.1)
CA 72-4 (U/mL)	1.8 (0.7–1100.0)
AFP (ng/mL)	2.9 (0.4–170736.0)
PSA (ng/mL)	0.8 (0.0–798.1)

* Presented with median instead of n, min–max instead of %,

† ECOG: Eastern Cooperative Oncology Group, CA: cancer antigen, AFP: alpha fetoprotein, PSA: prostate specific antigen, CEA: carcinoembryonic antigen.

**Table 2:** Treatment modalities of the CUP patients.

	N: 243 (%)
First-line chemotherapy	176 (72.4)
First-line chemotherapy regimens	
Gemcitabine/Cisplatin	87 (49.4)
Platin-containing regimens	34 (19.3)
Taxane-containing regimens	26 (14.8)
Other gemcitabine-containing regimens	12 (6.8)
Oxaliplatin containing regimens	12 (6.8)
Others	5 (2.8)
Second-line chemotherapy	50 (20.6)
Second-line chemotherapy regimens	
Oxaliplatin-containing regimens	21 (42.0)
Gemcitabine-containing regimens	12 (24.0)
Other platin-containing regimens	10 (20.0)
Others	7 (14.0)
Third-line chemotherapy	19 (7.8)
Third-line chemotherapy regimens	
Platin-containing regimens	5 (26.3)
Gemcitabine-containing regimens	3 (15.7)
Tekan-containing regimens	4 (21.0)
Others	7 (36.8)
Chemoembolization	18 (7.4)
Radiofrequency ablation	3 (1.2)

**Table 3:** Survival outcomes according to the first-line chemotherapy regimens.

	Median PFS time (months, 95% CI)	p-value	Median OS time (months, 95% CI)	p-value
First-line chemotherapy regimens		0.001		0.12
Gemcitabine/Cisplatin	6.8 (5.0–8.5)		10.3 (8.7–11.9)	
Platin-containing regimens	6.0 (4.1–8.0)		11.2 (2.3–20.1)	
Taxane-containing regimens	7.5 (4.1–10.9)		13.2 (2.6–23.8)	
Other gemcitabine-containing regimens	2.8 (1.9–3.6)		7.4 (1.7–13.1)	
Oxaliplatin containing regimens	3.3 (2.0–4.6)		13.8 (0.0–29.0)	
Others	2.10 (0.6–3.5)		3.0 (NA*)	

* NA: Not applicable, PFS: progression-free survival, OS: overall survival.

**Table 4:** Univariate and multivariate analysis of factors affecting OS.

	N: 243 (%)	Median-OS (months, 95% CI)	Univariate p-value	Multivariate HR (95% CI)	Multivariate p-value
Age group			0.57		
≤61	122 (50.2)	9.1 (6.9–11.3)			
>61	121 (49.8)	10.0 (6.2–13.8)			
Sex			0.11		
Male	150 (61.7)	9.1 (7.0–11.3)			
Female	93 (38.3)	9.5 (5.3–13.7)			
ECOG†			<0.001*	0.20 (0.10–0.40)	<0.001*
0–1	111 (45.7)	17.8 (14.7–20.9)			
≥2	132 (54.3)	3.8 (2.9–4.7)			
Cholestasis status			0.001*	0.48 (0.29–0.79)	0.004*
Yes	23 (9.5)	2.5 (0.0–5.5)			
No	220 (90.5)	9.9 (7.7–12.2)			
Histological type			0.68		
Adenocarcinoma	192 (79.0)	9.4 (7.1–11.8)			
Undifferentiated carcinoma	42 (17.3)	7.9 (4.3–11.5)			
SCC	9 (3.7)	9.9 (0.6–19.3)			
Number of metastatic sites			0.07		
1	68 (28.0)	12.5 (9.0–16.0)			
2	85 (35.0)	7.4 (4.5–10.2)			
≥3	90 (37.0)	6.8 (3.1–10.4)			
Lymph node metastasis			0.50		
Yes	198 (81.5)	9.4 (7.2–11.5)			
No	45 (18.5)	8.0 (2.1–13.9)			
Liver metastasis			0.05*	0.76 (0.53–1.07)	0.12
Yes	174 (71.6)	8.1 (5.9–10.4)			
No	69 (28.4)	10.0 (6.9–13.1)			
Lung metastasis			0.002*	0.69 (0.51–0.95)	0.02*
Yes	96 (39.5)	6.3 (4.7–7.9)			
No	147 (60.5)	10.4 (7.4–13.4)			
Bone metastasis			0.03*	1.02 (0.74–1.40)	0.86
Yes	71 (29.2)	5.8 (3.5–8.2)			
No	172 (70.8)	10.0 (7.9–12.1)			
Brain metastasis			0.92		
Yes	28 (11.5)	9.1 (3.8–14.4)			
No	215 (88.5)	9.1 (7.1–11.2)			
Peritoneal metastasis			0.89		
Yes	52 (21.4)	6.8 (3.9–9.6)			
No	191 (78.6)	9.5 (7.7–11.3)			
Adrenal gland metastasis			0.29		
Yes	24 (9.9)	5.0 (2.8–7.3)			
No	219 (90.1)	9.4 (7.5–11.4)			
First-line chemotherapy			0.03*	1.31 (0.84–2.03)	0.22
Yes	176 (72.4)	10.1 (8.3–11.9)			
No	67 (27.6)	4.2 (1.6–6.7)			
Second-line chemotherapy			<0.001*		<0.001*
Yes	50 (20.6)	16.8 (12.4–21.3)		1	
No	159 (65.4)	7.1 (5.2–9.1)		1.69 (1.14–2.49)	
Unknown	34 (14.0)	2.5 (0.5–4.6)		2.95 (1.78–4.89)	
Third-line chemotherapy			0.004*		0.71
Yes	19 (7.8)	27.4 (11.3–43.5)		1	
No	157 (64.6)	7.9 (5.1–10.7)		1.19 (0.60–2.32)	
Unknown	67 (27.6)	6.8 (4.4–9.2)		1.01 (0.45–2.26)	
Chemoembolization			0.26		
Yes	18 (7.4)	13.4 (10.4–16.3)			
No	225 (92.6)	7.9 (6.0–9.7)			
Radiofrequency ablation					
Yes	3 (1.2)	16.9 (5.7–28.1)	0.27		
No	239 (98.8)	8.6 (6.7–10.5)			

* Statistically significant,

† ECOG: Eastern Cooperative Oncology Group.

## References

[1] Pavlidis N, Fizazi K (2009). Carcinoma of unknown primary (CUP). Critical Reviews in Oncology/Hematology.

[2] Kwee TC, Kwee RM (2009). Combined FDG-PET/CT for the detection of unknown primary tumors: systematic review and meta-analysis. European Radiology.

[3] Van de Wouw A, Janssen-Heijnen M, Coebergh J, Hillen H (2002). Epidemiology of unknown primary tumours; incidence and population-based survival of 1285 patients in Southeast Netherlands, 1984–1992. European Journal of Cancer.

[4] Varadhachary GR, Raber MN (2014). Cancer of unknown primary site. New England Journal of Medicine.

[5] Hemminki K, Riihimäki M, Sundquist K, Hemminki A (2013). Site-specific survival rates for cancer of unknown primary according to location of metastases. International Journal of Cancer.

[6] Vikeså J, Møller AKH, Kaczkowski B, Borup R, Winther O (2015). Cancers of unknown primary origin (CUP) are characterized by chromosomal instability (CIN) compared to metastasis of know origin. BMC Cancer.

[7] Pavlidis N, Khaled H, Gaafar R (2015). A mini review on cancer of unknown primary site: a clinical puzzle for the oncologists. Journal of Advanced Research.

[8] Miller KD, Siegel RL, Lin CC, Mariotto AB, Kramer JL (2016). Cancer treatment and survivorship statistics, 2016. CA: A Cancer Journal for Clinicians.

[9] Varadhachary GR, Raber MN (2014). Carcinoma of unknown primary site. New England Journal of Medicine.

[10] Pavlidis N, Pentheroudakis G (2012). Cancer of unknown primary site. The Lancet.

[11] Hussien NM, Elsayed Z, Ibrahim DR, Eltabakh FM (2022). Predictors of survival of patients with cancer of unknown primary site: a retrospective study from two institutions in Egypt. Research in Oncology.

[12] Löffler H, Puthenparambil J, Hielscher T, Neben K, Krämer A (2014). Patients with cancer of unknown primary: a retrospective analysis of 223 patients with adenocarcinoma or undifferentiated carcinoma. Deutsches Ärzteblatt International.

[13] Raghav K, Mhadgut H, McQuade JL, Lei X, Ross A (2016). Cancer of unknown primary in adolescents and young adults: clinicopathological features, prognostic factors and survival outcomes. PLoS One.

[14] Algin E, Gumusay O, Yilmaz G, Buyukberber S, Coskun U (2016). Liver metastases from adenocarcinomas of unknown primary site: management and prognosis in 68 consecutive patients. Wiener klinische Wochenschrift.

[15] Pavlidis N, Briasoulis E, Hainsworth J, Greco F (2003). Diagnostic and therapeutic management of cancer of an unknown primary. European Journal of Cancer.

[16] Varadhachary GR, Talantov D, Raber MN, Meng C, Hess KR (2008). Molecular profiling of carcinoma of unknown primary and correlation with clinical evaluation. Journal of Clinical Oncology.

[17] Greco FA, Spigel DR, Yardley DA, Erlander MG, Ma X-J, Hainsworth JD (2010). Molecular profiling in unknown primary cancer: accuracy of tissue of origin prediction. The Oncologist.

[18] Hainsworth JD, Rubin MS, Spigel DR, Boccia RV, Raby S (2013). Molecular gene expression profiling to predict the tissue of origin and direct site-specific therapy in patients with carcinoma of unknown primary site: a prospective trial of the Sarah Cannon Research Institute. Journal of Clinical Oncology.

[19] Abbruzzese JL, Abbruzzese MC, Hess KR, Raber MN, Lenzi R (1994). Unknown primary carcinoma: natural history and prognostic factors in 657 consecutive patients. Journal of Clinical Oncology.

[20] Fernandez-Cotarelo MJ, Guerra-Vales JM, Colina F, de la Cruz J (2010). Prognostic factors in cancer of unknown primary site. Tumori Journal.

[21] Le Chevalier T, Cvitkovic E, Caille P, Harvey J, Contesso G (1988). Early metastatic cancer of unknown primary origin at presentation: a clinical study of 302 consecutive autopsied patients. Archives of Internal Medicine.

[22] Antuña Egocheaga A, López González ML, Lobo Fernández J, Fernández Bustamante J, Moris de la Tassa J (2002). Protocolo diagnóstico del cáncer de origen desconocido. Revisión de 157 casos. Anales de Medicina Interna.

[23] Pouesse D, Thezenas S, Culine S, Becht C, Senesse P (2005). Hepatic metastases from carcinomas of unknown primary site: experience of the Montpellier Cancer Center. Gastroenterologie Clinique et Biologique.

[24] Shaw P, Adams R, Jordan C, Crosby TDL (2007). A clinical review of the investigation and management of carcinoma of unknown primary in a single cancer network. Clinical Oncology.

[25] Chen K-W, Liu C-J, Lu H-J, Tzeng C-H, Liu J-H (2012). Evaluation of prognostic factors and the role of chemotherapy in unfavorable carcinoma of unknown primary site: a 10-year cohort study. BMC Research Notes.

[26] Culine S, Lortholary A, Voigt J-J, Bugat R, Théodore C (2003). Cisplatin in combination with either gemcitabine or irinotecan in carcinomas of unknown primary site: results of a randomized phase II study—trial for the French Study Group on Carcinomas of Unknown Primary (GEFCAPI 01). Journal of Clinical Oncology.

[27] Lee J, Hahn S, Kim D-W, Kim J, Kang S (2013). Evaluation of survival benefits by platinums and taxanes for an unfavourable subset of carcinoma of unknown primary: a systematic review and meta-analysis. British Journal of Cancer.

[28] Holtan SG, Steen PD, Foster NR, Erlichman C, Medeiros F (2012). Gemcitabine and irinotecan as first-line therapy for carcinoma of unknown primary: results of a multicenter phase II trial. PLoS One.

[29] Hainsworth JD, Spigel DR, Clark BL, Shipley D, Thompson DS (2010). Paclitaxel/carboplatin/etoposide versus gemcitabine/irinotecan in the first-line treatment of patients with carcinoma of unknown primary site: a randomized, phase III Sarah Cannon Oncology Research Consortium Trial. The Cancer Journal.

